# Case report: Cerebrotendinous xanthomatosis with a novel mutation in the *CYP27A1* gene mimicking behavioral variant frontotemporal dementia

**DOI:** 10.3389/fneur.2023.1131888

**Published:** 2023-03-07

**Authors:** Min Young Chun, Nam Jin Heo, Sang Won Seo, Hyemin Jang, Yeon-Lim Suh, Ja-Hyun Jang, Young-Eun Kim, Eun-Joo Kim, So Young Moon, Na-Yeon Jung, Sun Min Lee, Hee Jin Kim

**Affiliations:** ^1^Department of Neurology, Samsung Medical Center, Sungkyunkwan University School of Medicine, Seoul, Republic of Korea; ^2^Department of Neurology, Yonsei University College of Medicine, Seoul, Republic of Korea; ^3^Department of Neurology, Yongin Severance Hospital, Yonsei University Health System, Yongin, Republic of Korea; ^4^Department of Health Sciences and Technology, SAIHST, Sungkyunkwan University, Seoul, Republic of Korea; ^5^Department of Digital Health, SAIHST, Sungkyunkwan University, Seoul, Republic of Korea; ^6^Alzheimer's Disease Convergence Research Center, Samsung Medical Center, Seoul, Republic of Korea; ^7^Department of Pathology, Samsung Medical Center, Sungkyunkwan University School of Medicine, Seoul, Republic of Korea; ^8^Department of Laboratory Medicine and Genetics, Samsung Medical Center, Sungkyunkwan University School of Medicine, Seoul, Republic of Korea; ^9^Departments of Laboratory Medicine, Hanyang University College of Medicine, Seoul, Republic of Korea; ^10^Department of Neurology, Pusan National University Hospital, Pusan National University School of Medicine and Medical Research Institute, Busan, Republic of Korea; ^11^Department of Neurology, Ajou University School of Medicine, Suwon, Republic of Korea; ^12^Department of Neurology, Pusan National University Yangsan Hospital, Research Institute for Convergence of Biomedical Science and Technology, Yangsan, Republic of Korea

**Keywords:** behavioral variant frontotemporal dementia, cerebrotendinous xanthomatosis, *CYP27A1* gene mutation, novel likely pathogenic variant, case report

## Abstract

**Background:**

Cerebrotendinous xanthomatosis (CTX) is a rare autosomal recessive lipid storage disease caused by a mutation in the *CYP27A1* gene. Due to the disruption of bile acid synthesis leading to cholesterol and cholestanol accumulation, CTX manifests as premature cataracts, chronic diarrhea, and intellectual disability in childhood and adolescence. This report presents a case of CTX with an unusual phenotype of behavioral variant frontotemporal dementia (bvFTD) in middle age.

**Case presentation:**

A 60-year-old woman presented with behavioral and personality changes. She showed disinhibition, such as hoarding and becoming aggressive over trifles; compulsive behavior, such as closing doors; apathy; and dietary change. The patient showed a progressive cognitive decline and relatively sparing memory and visuospatial function. She had hyperlipidemia but no family history of neurodegenerative disorders. Initial fluid-attenuated inversion recovery (FLAIR) images showed a high signal in the periventricular area, and brain spectroscopy showed hypoperfusion in the frontal and temporal lobes, mimicking bvFTD. However, on physical examination, xanthomas were found on both the dorsum of the hands and the Achilles tendons. Hyperactive deep tendon reflexes in the bilateral biceps, brachioradialis, and knee and positive Chaddock signs on both sides were observed. Four years later, FLAIR images showed symmetrical high signals in the bilateral dentate nuclei of the cerebellum. Her serum cholestanol (12.4 mg/L; normal value ≤6.0) and 7α,12α-dihydroxycholest-4-en-3-one (0.485 nmol/mL; normal value ≤0.100) levels were elevated. A novel likely pathogenic variant (c.1001T>A, p.Met334Lys) and a known pathogenic variant (c.1420C>T, p.Arg474Trp) of the *CYP27A1* gene were found in trans-location. The patient was diagnosed with CTX and prescribed chenodeoxycholic acid (750 mg/day).

**Conclusions:**

This report discusses the case of a middle-aged CTX patient with an unusual phenotype of bvFTD. A novel likely pathogenic variant (c.1001T>A, p.Met334Lys) was identified in the *CYP27A1* gene. Early diagnosis is important because supplying chenodeoxycholic acid can prevent CTX progression.

## Introduction

Cerebrotendinous xanthomatosis (CTX, OMIM: 213700) is an autosomal recessive lipid storage disease caused by a mutation in the *CYP27A1* gene (chromosome 2q33-qter) (1). Mutations in the gene encoding the mitochondrial enzyme sterol 27-hydroxylase (CYP27) can lead to decreased bile acid synthesis, increased cholestanol production, and sterol accumulation in multiple systems, including the nervous system, tendons, and eye lenses (2). It is a rare disease with an incidence of ~5 per 100,000 people worldwide, and 10 cases have been reported in South Korea (3–10). This report discusses a case of CTX with an unusual phenotype of behavioral variant frontotemporal dementia (bvFTD).

## Case description

A 60-year-old woman visited the Department of Neurology, Samsung Medical Center in South Korea because of progressive abnormal behavior, personality change, and cognitive decline over the past 4 years. At the age of 56, she showed disinhibition, such as hoarding plastic bottles or paper cups and becoming aggressive over trifles. She also showed compulsive behavior, such as closing doors; apathy; and dietary change. The patient showed a progressive cognitive decline and relatively sparing memory and visuospatial function. However, at the age of 58, she started to show memory impairment as she could not remember where she put her money. She also showed visuospatial dysfunction as she got lost in her neighborhood and could not find her car in a parking lot. At the age of 60, disinhibition and apathy worsened along with increased appetite. In addition, she complained of nonspecific dizziness and unstable gait. She had a history of hyperlipidemia and no family history of neurological diseases in her first- or second-degree relatives.

**Figure 1:**
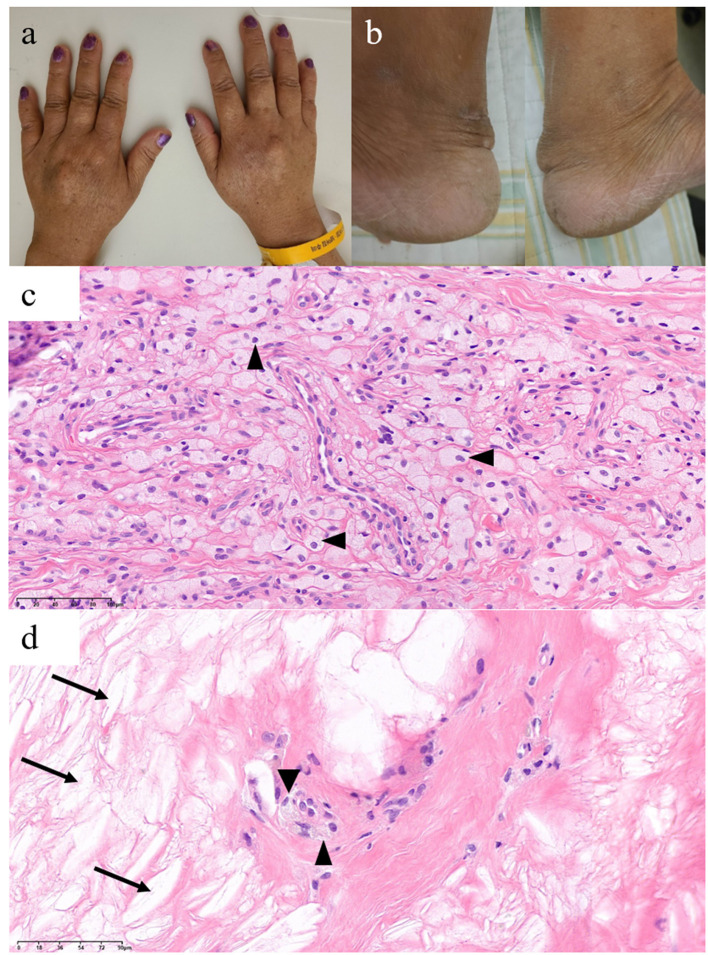
Xanthomas findings of this patient with cerebrotendinous xanthomatosis. Xanthomas on bilateral **(a)** dorsum of the hands and **(b)** Achilles tendons. Histological findings of the dorsum of the hand **(c)** and Achilles tendon **(d)** xanthomas show foamy macrophages (arrowheads) and lipid crystal clefts (arrows).

On neuropsychological tests, her Mini-Mental State Examination score was 22, her clinical dementia rating score was 1, and the detailed results revealed global cognitive impairment (Table 1). On neurological examination, she showed hyperactive deep tendon reflexes in the bilateral biceps, brachioradialis, and knee and positive Chaddock signs on both sides. Although no gross gait abnormalities or ataxia were observed, she showed bilateral sway on tandem gait. Upon physical examination, xanthomas were found in the bilateral dorsum of the hands and the Achilles tendons (Figures 1a, b). A skin biopsy revealed diffuse infiltration of foamy macrophages in the dermis, and a tendon biopsy revealed numerous lipid crystal clefts and a small number of foamy macrophages (Figures 1c, d).

**Table 1:** The neuropsychological test results.

**Age**	**60 years old (before treatment)**		**61 years old (after treatment)**	
**Domain (highest possible score)**	**Raw score**	**z-score**	**Raw score**	**z-score**
**Attention**				
Digit span forward (9)	6	0.04	6	0.04
Digit span backward (8)	3	−0.93	3	−0.93
**Language**				
Korean version of the Boston naming Test (60)	37^*^	−2.05	35^*^	−2.39
**Visuospatial function**				
Rey-Osterrieth Complex Figure Test (36)	23.5^*^	−2.93	27^*^	−1.81
**Memory**				
Seoul verbal learning test				
Immediate recall (1st, 2nd, 3rd free recall trials: 12 + 12 + 12 = 36)	12^*^	−1.97	19	−0.39
Delayed recall (12)	1^*^	−2.52	3^*^	−1.65
Recognition score (24)	13^*^	−4.32	19^*^	−1.11
**Rey-Osterrieth complex figure test**				
Immediate recall (36)	0^*^	−2.43	5^*^	−1.65
Delayed recall (36)	4^*^	−1.89	3^*^	−2.05
Recognition score (24)	17^*^	−1.61	10	0.04
**Frontal/executive function**				
Controlled oral word association test				
Semantic, animal (20)	6^*^	−2.43	9^*^	−1.68
Semantic, supermarket (20)	5^*^	−2.33	11^*^	−1.23
Phonemic, sum of scores from 3 alphabets (45)	8^*^	−1.90	3^*^	−2.47
**Stroop test**				
Word reading (112)	111		17	
Color reading (112)	52^*^	−2.06	73	−0.82
**General cognition**				
Mini-mental state examination (30)	22^*^	−3.69	26^*^	−1.23
**Global severity scales**				
Clinical dementia rating (sum of boxes)	1 (6)		0.5 (4)	
Global deterioration scale	5		4	
Neuropsychiatric inventory	38/144		9/144	

* Below −1.0 standard deviation of age- and education-matched norms derived from the Seoul Neuropsychological Screening Battery (11). The source for all tests is the Seoul Neuropsychological Screening Battery (11).

**Figure 2:**
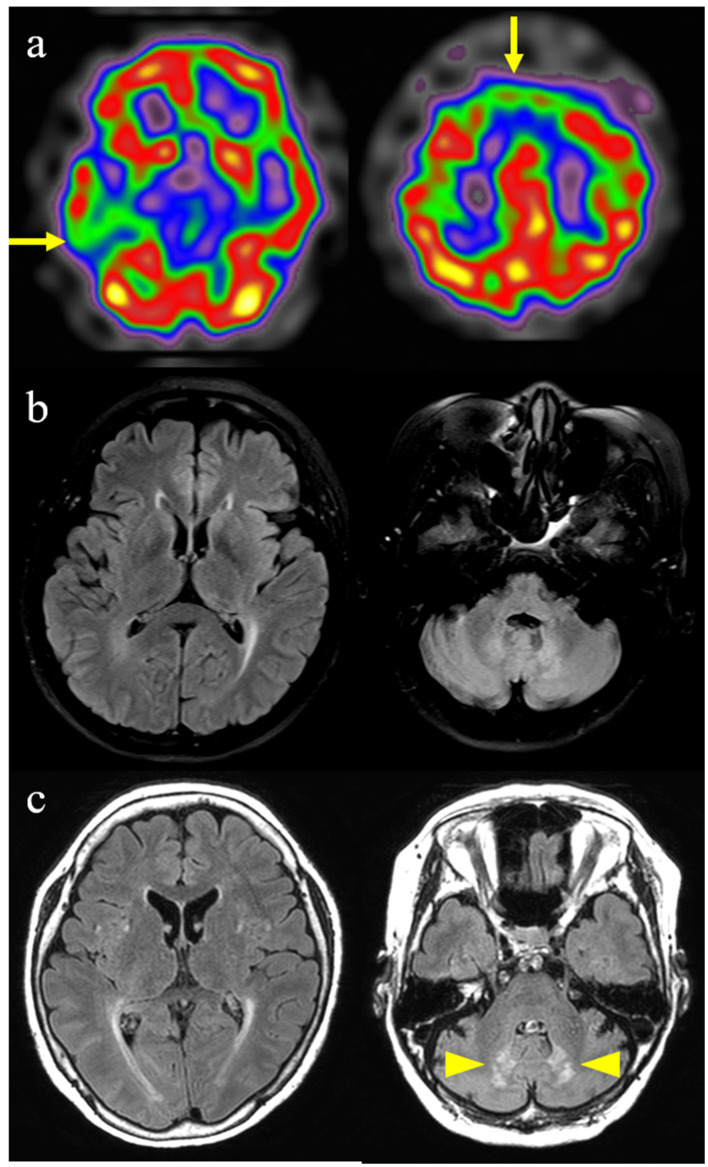
Brain imaging findings of this patient with cerebrotendinous xanthomatosis. **(a)** Brain spectroscopy shows hypoperfusion in bilateral frontal and right temporal lobes (arrows). **(b)** Initial fluid-attenuated inversion recovery image shows slight periventricular white matter changes. **(c)** Follow-up fluid-attenuated inversion recovery image performed 4 years later shows the progression of periventricular white matter changes and newly developed symmetrical hyperintense lesions in the dentate nucleus of the cerebellum (arrowheads).

Brain spectroscopy performed at 56 years of age showed hypoperfusion in the bilateral frontal and right temporal lobes (Figure 2a). A brain magnetic resonance imaging (MRI) performed during the same period showed slight periventricular white matter changes on T2 FLAIR images, but no other specific abnormalities were observed (Figure 2b). An ^18^F-flutemetamol PET scan was interpreted as negative for amyloid. Her clinical phenotype and spectroscopy findings corresponded to probable bvFTD based on Rascovsky criteria for bvFTD (12).

However, follow-up T2 FLAIR images, performed at the age of 60 years, demonstrated progression of periventricular white matter changes and newly developed symmetrical hyperintense lesions in the dentate nucleus of the cerebellum (Figure 2c). Although no evidence of peripheral neuropathy was found in the nerve conduction study (NCS), her posterior tibial somatosensory evoked potential (SSEP) and visual evoked potential tests (VEP) were abnormal. Bone mineral osteodensitometry revealed the presence of osteoporosis. Ophthalmic examination revealed age-related macular degeneration with no cataracts or retinal invasion.

CTX was suspected based on xanthomas and follow-up brain MRI findings; thus, serum cholestanol and related gene tests were performed. Serum cholestanol (12.4 mg/L; normal value ≤6.0) and 7α,12α-dihydroxycholest-4-en-3-one (0.485 nmol/mL; normal value ≤0.100) levels were elevated. The serum levels of very-long-chain fatty acids for X-linked adrenoleukodystrophy and arylsulfatase A for metachromatic leukodystrophy were within the normal range. In genetic analysis with whole exome sequencing, a likely pathogenic variant (LPV) (NM_00784.4:c.1001T>A, p.Met334Lys) and a pathogenic variant (PV, NM_000784.4: c.1420C>T, p.Arg474Trp) were found in the *CYP27A1* gene, which was confirmed to be translocated via genetic testing of her daughter and son, who carried each variant as heterozygous. The c.1001T>A (p.Met334Lys) had not been reported previously and the c.1420C>T (p.Arg474Trp) has been observed in several CTX patients and classified as pathogenic in ClinVar. (ClinVar accession number as VCV000004259.12) (13–18). The patient was finally diagnosed with CTX and was prescribed with chenodeoxycholic acid (750 mg/day). Eight months after the treatment, her disinhibition symptoms decreased. In neuropsychological tests, she showed improvements in visuospatial function, memory, frontal/executive function, general cognition, and global severity scales (Table 1).

## Discussion

This report discusses the case of a middle-aged CTX patient with an unusual phenotype of bvFTD, confirmed by a novel likely pathogenic and a known pathogenic variant, as compound heterozygous variants, (c.[1001T>A];[1420C>T], p.[Met334Lys];[Arg474Trp]) in the *CYP27A1* gene with elevated serum cholestanol and 7α,12α-dihydroxycholest-4-en-3-one levels.

Our CTX case is unique in that disease onset occurred during middle age and that the patient showed an unusual bvFTD phenotype. CTX is usually found in adolescence or early adulthood and is characterized by premature bilateral cataracts, chronic diarrhea in childhood, premature atherosclerosis, tendon xanthoma, and progressive neurological dysfunction (cerebellar and pyramidal signs, intellectual disability, peripheral neuropathy, and seizures) (19). However, our patient had no such premature symptoms and showed slow progressive abnormal behavior during late middle age. Her slowly progressive symptoms of disinhibition, apathy, compulsive behavior, and dietary changes and her frontotemporal hypoperfusion met the clinical criteria for probable bvFTD (12). There have been two rare case reports of CTX presenting with frontal dysfunction in adults. A 44-year-old woman with the FTD phenotype was reported in Japan (20). This patient had increased serum levels of cholestanol with a heterozygous mutation in the *CYP27A1* gene. Another 53-year-old man with the FTD phenotype of CTX was reported in the United States. This patient was compound heterozygous for two mutations in *CYP27A1* (NM_000784.3 (CYP27A1): a missense mutation of 1016C > T on one allele and a 1435C > G mutation on the other allele) (21). The two previously reported cases, along with our case, suggest that the middle-age-onset bvFTD phenotype might be a subtype of CTX.

The FTD phenotype of CTX can be explained by diffuse white matter pathology and neuronal loss, shown as symmetric high-intensity on brain MR T2-weighted images in the periventricular cerebral white matter as well as diffuse atrophy. White matter pathology may result from the disproportionate incorporation of cholesterol into the glial cell membrane and alterations in myelin lipid composition (22, 23). Also, intracerebral lipid deposition associated with xanthomas and local inflammatory responses can damage myelinated axons, gray matter formation, neuronal cell bodies, and neuronal integrity (19, 24), leading to neuronal loss and deterioration in behavior and cognition.

Several differential diagnoses should be considered when assessing patients with xanthomas and symmetric lesions in the dentate nuclei of the cerebellum. Tendonous xanthomas need to be differentiated from other hereditary diseases, such as familial hypercholesterolemia and sitosterolemia (1). Familial hypercholesterolemia, the most common cause of tendon xanthomas, is an autosomal dominant disorder that leads to increased low-density lipoprotein cholesterol levels, but with normal cholestanol levels (25). While familial hypercholesterolemia usually manifests as intertriginous xanthomas in children, sitosterolemia and CTX manifest as tendonous xanthomas in adults (1, 26). Sitosterolemia can be differentiated from CTX by the absence of neurological symptoms and premature cataracts (27). Furthermore, hyperintensities of dentate nuclei on MRI should be differentiated from other diseases, including metronidazole toxicity, lead poisoning, maple syrup urine disease, and progressive multifocal leukoencephalopathy caused by the JC virus (28). These disorders manifest as acute encephalopathy rather than the insidious onset seen in CTX. Thus, if progressive bvFTD symptoms appear in adults with xanthoma or a characteristic appearance of dentate in MRI, the possibility of CTX should be considered; the serum cholestanol and 7α,12α-dihydroxycholest-4-en-3-one levels should be further evaluated, and mutations in the *CYP27A1* gene (1) should be searched for.

In addition, the patient showed abnormalities in SSEP and VEP, which supports the diagnosis of CTX (1, 29–31). Our patient was also revealed to have osteoporosis and ocular abnormalities, which are widely described as common manifestations in CTX patients (1, 32, 33).

Our patient was confirmed to have CTX based on biochemical and genetic tests. Biochemical tests showed increased levels of cholestanol and 7α,12α-dihydroxycholest-4-en-3-one, which is a highly sensitive metabolic biomarker of CTX (34). Genetic tests identified an LPV (c.1001T>A, p.Met334Lys) and a PV (c.1420C>T, p.Arg474Trp) in the *CYP27A1* gene in trans. The variant c.1420C>T (p.Arg474Trp) has been found in several CTX patients as homozygous or compound heterozygous (13–18) and classified as pathogenic in ClinVar (accession number: VCV000004259.12). Although c.1001T>A (p.Met334Lys) has not been reported as a cause of CTX, we suggest the variant is likely pathogenic based on the following evidence: (i) The p.Met334Lys is located in a chemical substrate binding site in the P450 domain, which is a critical and well-established functional domain. In particular, the region from the 330th to the 345th amino acid is clustered by the binding site without any known benign variant (PM1). (ii) The p.Met334Lys is absent in the population database (gnomAD, https://gnomad.broadinstitute.org/, accessed on 27, Jan.2023) (PM2). (iii) The p.Met334Lys was located in *trans* with the PV, c.1420C>T (PM3), and the patient's phenotype and biochemical test results were highly specific for CTX (PP4). Therefore, we classified the variant as likely pathogenic with three moderate evidences of pathogenicity.

In conclusion, CTX is an underdiagnosed disease, and the phenotype is often incomplete. Progressive dementia can be the only neuropsychiatric sign associated with CTX (21). Early recognition and intervention of CTX are important because treatment with chenodeoxycholic acid reverses metabolic abnormalities and prevents or ameliorates nervous system dysfunction. Therefore, we suggest that the diagnosis of CTX should be considered in patients with progressive dementia and xanthoma, even in the absence of premature CTX symptoms.

## Data availability statement

The datasets presented in this article are not readily available because of ethical and privacy restrictions. Requests to access the datasets should be directed to the corresponding author.

## Ethics statement

The studies involving human participants were reviewed and approved by Institutional review board of Samsung Medical Center. The patients/participants provided their written informed consent to participate in this study. Written informed consent was obtained from the participant/patient(s) for the publication of this case report.

## Author contributions

MC interpreted the patient data regarding cerebrotendinous xanthomatosis disease and was a major contributor to the writing of the manuscript. E-JK, SM, N-YJ, and SL collected data and helped to draft the manuscript. NH, SS, and HJ reviewed the patient data and manuscript. Y-LS performed a pathological examination of the skin. J-HJ and Y-EK performed the genetic analysis of the patient and the next of kin and interpreted the results. HK supervised and reviewed the manuscript. All authors contributed to the article and approved the submitted version.
